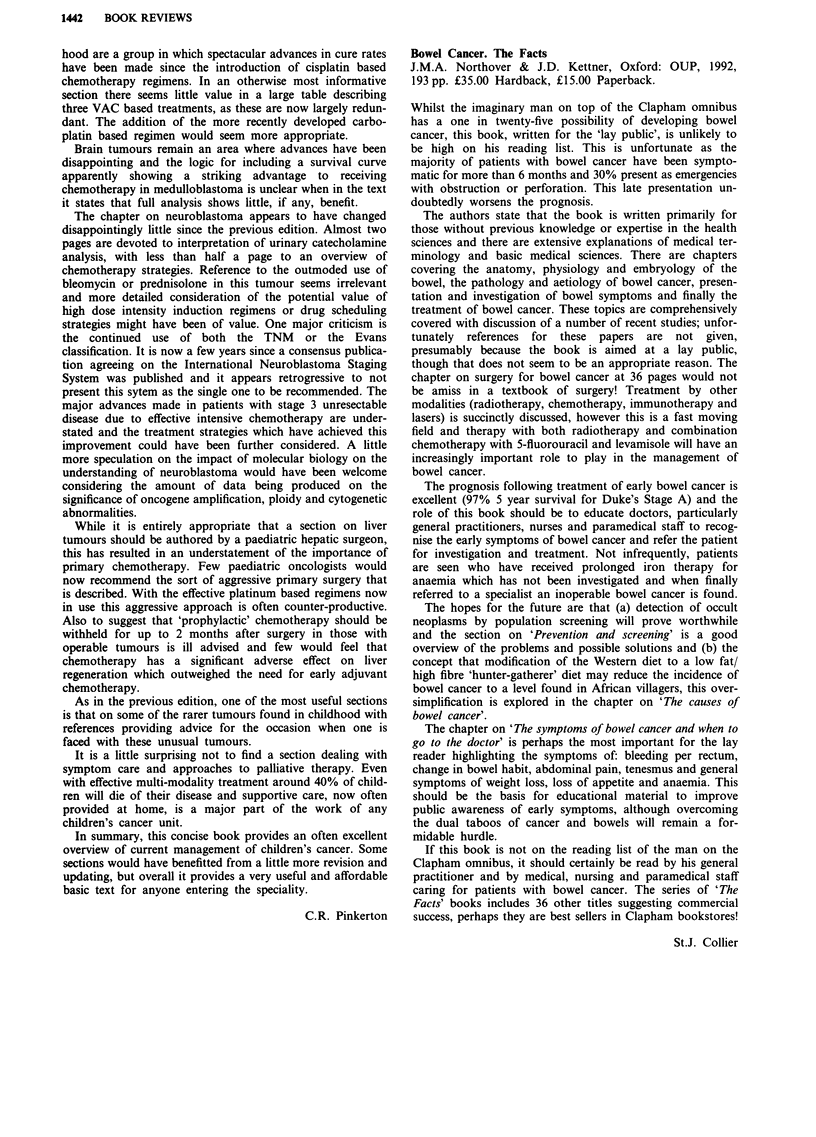# Bowel Cancer. The Facts

**Published:** 1993-06

**Authors:** J. Collier


**Bowel Cancer. The Facts**

J.M.A. Northover & J.D. Kettner, Oxford: OUP, 1992, 193 pp. £35.00 Hardback, £15.00 Paperback.

Whilst the imaginary man on top of the Clapham omnibus has a one in twenty-five possibility of developing bowel cancer, this book, written for the 'lay public', is unlikely to be high on his reading list. This is unfortunate as the majority of patients with bowel cancer have been symptomatic for more than 6 months and 30% present as emergencies with obstruction or perforation. This late presentation undoubtedly worsens the prognosis.

The authors state that the book is written primarily for those without previous knowledge or expertise in the health sciences and there are extensive explanations of medical terminology and basic medical sciences. There are chapters covering the anatomy, physiology and embryology of the bowel, the pathology and aetiology of bowel cancer, presentation and investigation of bowel symptoms and finally the treatment of bowel cancer. These topics are comprehensively covered with discussion of a number of recent studies; unfortunately references for these papers are not given, presumably because the book is aimed at a lay public, though that does not seem to be an appropriate reason. The chapter on surgery for bowel cancer at 36 pages would not be amiss in a textbook of surgery! Treatment by other modalities (radiotherapy, chemotherapy, immunotherapy and lasers) is succinctly discussed, however this is a fast moving field and therapy with both radiotherapy and combination chemotherapy with 5-fluorouracil and levamisole will have an increasingly important role to play in the management of bowel cancer.

The prognosis following treatment of early bowel cancer is excellent (97% 5 year survival for Duke's Stage A) and the role of this book should be to educate doctors, particularly general practitioners, nurses and paramedical staff to recognise the early symptoms of bowel cancer and refer the patient for investigation and treatment. Not infrequently, patients are seen who have received prolonged iron therapy for anaemia which has not been investigated and when finally referred to a specialist an inoperable bowel cancer is found.

The hopes for the future are that (a) detection of occult neoplasms by population screening will prove worthwhile and the section on '*Prevention and screening*' is a good overview of the problems and possible solutions and (b) the concept that modification of the Western diet to a low fat/high fibre 'hunter-gatherer' diet may reduce the incidence of bowel cancer to a level found in African villagers, this over-simplification is explored in the chapter on '*The causes of bowel cancer*'.

The chapter on '*The symptoms of bowel cancer and when to go to the doctor*' is perhaps the most important for the lay reader highlighting the symptoms of: bleeding per rectum, change in bowel habit, abdominal pain, tenesmus and general symptoms of weight loss, loss of appetite and anaemia. This should be the basis for educational material to improve public awareness of early symptoms, although overcoming the dual taboos of cancer and bowels will remain a formidable hurdle.

If this book is not on the reading list of the man on the Clapham omnibus, it should certainly be read by his general practitioner and by medical, nursing and paramedical staff caring for patients with bowel cancer. The series of '*The Facts*' books includes 36 other titles suggesting commercial success, perhaps they are best sellers in Clapham bookstores!

St.J. Collier